# Molecular and Microenvironmental Mechanisms of Malignant Transformation in Benign Salivary Gland Tumors: Implications for Oral Squamous Cell Carcinoma

**DOI:** 10.3390/diagnostics16060898

**Published:** 2026-03-18

**Authors:** Panagiotis Giasimakopoulos, Danai Mylona, Aggelos Diafas, Ioannis Stamoulopoulos, Konstantinos Markou

**Affiliations:** 1 Department of Oral and Maxillofacial Surgery, Aristotle University of Thessaloniki, 54124 Thessaloniki, Greece; 2 Postgraduate Program in Orthodontics, European University Cyprus, 2404 Nicosia, Cyprus; 3 School of Medicine, Aristotle University of Thessaloniki, 54124 Thessaloniki, Greece; 4 Department of Otorhinolaryngology—Head and Neck Surgery, School of Medicine, Aristotle University of Thessaloniki, 54124 Thessaloniki, Greece

**Keywords:** salivary gland tumors, malignant transformation, pleomorphic adenoma, carcinoma ex pleomorphic adenoma, molecular biomarkers, angiogenesis, tumor microenvironment, epigenetic alterations, oral squamous cell carcinoma

## Abstract

Malignant transformation of benign salivary gland tumors represents a critical biological process that provides valuable insights into head and neck carcinogenesis, with potential implications for oral squamous cell carcinoma (OSCC). Understanding the molecular, epigenetic, and microenvironmental mechanisms underlying this transition is essential for improving early diagnosis, risk stratification, and personalized management strategies. This study presents a comprehensive narrative review of the current literature focusing on benign salivary gland tumors with malignant potential, particularly pleomorphic adenoma and carcinoma ex pleomorphic adenoma, emphasizing molecular alterations, angiogenesis, and tumor microenvironment dynamics. A structured literature search was conducted across major biomedical databases, including PubMed and Scopus, selecting studies that addressed genetic rearrangements, epigenetic modifications, histopathological features, and clinical connections of malignant transformation. The findings highlight recurrent genetic alterations such as *PLAG1* and *HMGA2* rearrangements, *TP53* mutations, and *ERBB2* overexpression, along with epigenetic dysregulation through CpG island hypermethylation. Enhanced angiogenesis, marked by increased expression of CD105 and vascular endothelial growth factor, as well as a “cold” immune microenvironment, emerged as key contributors to tumor progression. These mechanisms demonstrate significant overlap with pathways implicated in OSCC development. Benign salivary gland tumors represent a valuable model for studying malignant transformation in head and neck oncology. Interpreting shared molecular and microenvironmental pathways may facilitate the identification of novel biomarkers and support the development of personalized diagnostic and therapeutic approaches for OSCC.

## 1. Introduction

Salivary gland tumors constitute a heterogeneous group of neoplasms characterized by diverse histological patterns, biological behavior, and clinical outcomes [1,2,3]. Although most salivary gland tumors are benign, a subset demonstrates the potential for malignant transformation, a process associated with increased morbidity, therapeutic complexity, and poorer prognosis [4]. Among benign salivary gland tumors, pleomorphic adenoma represents the most common entity and accounts for the majority of cases undergoing malignant transformation, most frequently evolving into carcinoma ex pleomorphic adenoma (CXPA) [5].

Malignant transformation is increasingly regarded as a multistep process driven by cumulative genetic, epigenetic, and microenvironmental alterations [6]. Molecular studies indicate that recurrent chromosomal rearrangements involving *PLAG1* and *HMGA2* represent early oncogenic events in pleomorphic adenoma tumorigenesis and may persist across progression, supporting a clonal continuum from benign to malignant disease [7,8,9,10]. As tumors evolve, additional genomic abnormalities—including alterations affecting key tumor suppressor and growth signaling pathways—have been associated with aggressive phenotypes and high-grade malignant transformation in CXPA [11].

Epigenetic dysregulation also plays an important role in tumor progression. Aberrant DNA methylation affecting tumor suppressor genes, including *CDKN2A (p16)* and *RASSF1A*, has been reported in salivary gland tumors and linked to loss of cell-cycle control and malignant progression [12,13,14,15].

In addition to tumor-intrinsic molecular changes, the tumor microenvironment has emerged as a key contributor to malignant progression. Angiogenesis, reflected by increased microvessel density and upregulation of neovascular markers such as CD105 (endoglin), has been associated with invasive behavior and metastatic potential in salivary gland neoplasms [16,17]. Moreover, VEGF signaling is a central driver of tumor angiogenesis and is mechanistically linked to vascular expansion and progression across multiple malignancies [18]. Beyond vascular remodeling, alterations in immune surveillance—including reduced immune-mediated tumor control—may facilitate immune escape and tumor evolution, with emerging evidence implicating immune surveillance and dendritic-cell-related mechanisms in malignant transformation and CXPA biology [19,20].

Despite advances in molecular pathology, the diagnosis of malignant transformation remains challenging because clinical and radiological findings may overlap between benign and malignant lesions [21]. Histopathological evaluation remains the definitive method for diagnosis, while molecular profiling and immunohistochemical biomarkers are increasingly investigated for their potential utility in risk stratification and prognostic assessment [22,23,24]. The aim of this review is to summarize current evidence regarding the clinical, histopathological, molecular, and microenvironmental mechanisms involved in the malignant transformation of benign salivary gland tumors, with particular emphasis on pleomorphic adenoma and carcinoma ex pleomorphic adenoma [5,21].

## 2. Materials and Methods

### 2.1. Study Design

This manuscript was designed as a narrative literature review based exclusively on previously published studies focusing on the malignant transformation of benign salivary gland tumors, with particular emphasis on molecular, genetic, histopathological, and microenvironmental mechanisms that may contribute to carcinogenesis. This study aimed to synthesize current evidence relevant to diagnostic refinement and clinical risk stratification in salivary gland neoplasia, in line with contemporary oncologic and head and neck research frameworks and current tumor classification systems [25,26,27,28].

### 2.2. Literature Search Strategy

A structured literature search was conducted using major biomedical databases, including PubMed/MEDLINE, Scopus, and Web of Science. The literature search included studies published from January 2015 to January 2024. Additional relevant sources were identified through manual screening of reference lists from key articles and review papers.

The literature search initially identified 114 records across the selected databases. After title and abstract screening, 82 studies were considered potentially relevant. Following the removal of duplicate or non-relevant records, 65 full-text articles were evaluated and included in the qualitative synthesis. Among these, 25 studies provided detailed data specifically addressing malignant transformation mechanisms in benign salivary gland tumors.

The search strategy combined Medical Subject Headings (MeSH) terms and free-text keywords related to salivary gland neoplasms and malignant transformation. The principal search terms included combinations of: “salivary gland tumors”, “benign salivary gland neoplasms”, “malignant transformation”, “carcinoma ex pleomorphic adenoma”, “*PLAG1*”, “*HMGA2*”, “epigenetic alterations”, “angiogenesis”, “tumor microenvironment”, and “molecular biomarkers”.

### 2.3. Eligibility Criteria

Articles were included if they met the following criteria:

- Published in peer-reviewed scientific journals
- Written in English
- Focused on benign salivary gland tumors and/or their malignant transformation
- Provided clinical, histopathological, molecular, or genetic data relevant to malignant progression

Case reports, case series, retrospective and prospective studies, and authoritative review articles were considered eligible due to the relative rarity of malignant transformation in salivary gland tumors [25].

Exclusion criteria included non-English publications, studies lacking sufficient pathological or molecular characterization, and articles not directly related to salivary gland tumor biology.

### 2.4. Data Extraction and Analysis

Data were extracted based on predefined thematic categories, including tumor type, reported frequency of malignant transformation, genetic and epigenetic alterations, angiogenic markers, characteristics of the immune microenvironment, and clinical behavior.

Particular emphasis was placed on well-established molecular pathways, reproducible histopathological findings, and emerging biomarkers associated with malignant progression of pleomorphic adenoma and related salivary gland tumors [25,26].

The extracted data were synthesized qualitatively, allowing for an integrative interpretation of the mechanisms underlying malignant transformation rather than quantitative meta-analysis, due to heterogeneity in study designs and reported outcomes.

No new experimental data were generated or analyzed in this study.

### 2.5. Ethical Considerations

As this study was based exclusively on previously published data, no ethical approval or informed consent was required.

## 3. Results

The results of the present narrative review are organized into thematic subsections summarizing the main findings reported in the literature regarding the malignant transformation of benign salivary gland tumors. Emphasis is placed on epidemiological patterns, molecular and genetic alterations, histopathological changes, and microenvironmental factors associated with malignant progression.

### 3.1. Frequency of Malignant Transformation in Benign Salivary Gland Tumors

The reported frequency of malignant transformation varies significantly among different benign salivary gland tumors.

Pleomorphic adenoma (PA) is consistently reported in the literature as the benign salivary gland tumor with the highest propensity for malignant transformation, with estimated rates ranging from 1.5% to 12%. This variability appears to be influenced by several factors, including tumor duration, history of recurrence, and differences in study design and diagnostic criteria [29,30]. The most frequently encountered malignant entity arising from pleomorphic adenoma is carcinoma ex pleomorphic adenoma (CXPA), which represents the predominant form of malignant transformation associated with PA [30].

In contrast, Warthin tumor exhibits an exceptionally low risk of malignant transformation, generally estimated to be less than 1%. The majority of reported malignant cases occurring in association with Warthin tumors are considered coincidental synchronous or metachronous malignancies rather than true malignant transformation of the benign lesion itself [31,32,33].

Other benign salivary gland tumors, including basal cell adenoma, oncocytoma, and lipoma, have only rarely been linked to malignant change. Such associations are largely confined to isolated case reports, underscoring the exceedingly low malignant potential of these entities [34].

These findings indicate that tumor histology and biological behavior are key determinants of malignant potential in salivary gland neoplasia.

### 3.2. Molecular and Genetic Alterations Associated with Malignant Transformation

#### Gene Rearrangements and Mutations

Multiple studies have emphasized the contribution of recurrent genetic alterations to the malignant progression of benign salivary gland tumors.

Rearrangements involving the *PLAG1* gene are frequently identified in pleomorphic adenomas and are regarded as early oncogenic events that are retained in a substantial proportion of cases progressing to carcinoma ex pleomorphic adenoma (CXPA), supporting their role in tumor initiation rather than late malignant transformation [35,36].

Similarly, alterations affecting *HMGA2* represent another key molecular event in pleomorphic adenoma tumorigenesis. Overexpression or structural alterations of *HMGA2* have been documented in both benign and malignant salivary gland tumors, indicating that these changes may contribute to early tumor development and subsequent progression [37,38].

More recently, specific gene fusions such as *HMGA2::WIF1* were reported in salivary gland neoplasms and are associated with tumors demonstrating loss of the classical biphasic histological architecture characteristic of pleomorphic adenoma. These rearrangements appear to correlate with increased malignant potential and more aggressive tumor phenotypes [39].

In addition to these early genetic events, further molecular alterations appear to contribute to tumor progression and aggressiveness. Mutations involving *TP53* and amplification of *ERBB2* (HER2/neu) are predominantly observed in high-grade CXPA and are associated with invasive growth patterns, increased proliferative activity, and unfavorable clinical outcomes [40,41].

**Figure 1:**
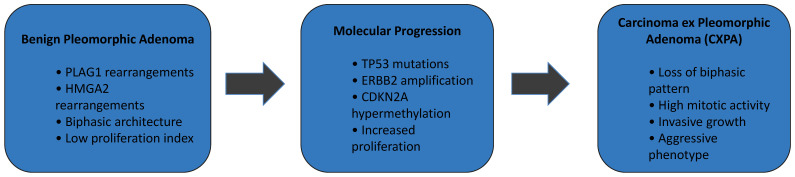
Multistep molecular model of malignant transformation in pleomorphic adenoma. Pleomorphic adenoma (PA) is characterized by recurrent *PLAG1* and *HMGA2* rearrangements and a biphasic epithelial–myoepithelial architecture with low proliferative activity. Accumulation of additional genetic and epigenetic alterations, including *TP53* mutations, *ERBB2* amplification, and *CDKN2A* hypermethylation, promotes molecular progression. These events culminate in carcinoma ex pleomorphic adenoma (CXPA), which demonstrates loss of biphasic morphology, increased mitotic activity, invasive growth, and aggressive clinical behavior. This figure was created by the authors for this study.

These molecular findings suggest a multistep carcinogenic process, with early driver events followed by secondary mutations promoting malignancy. The multistep molecular evolution from pleomorphic adenoma to carcinoma ex pleomorphic adenoma is summarized in Figure 1.

To contextualize the molecular mechanisms involved in carcinoma ex pleomorphic adenoma within the broader landscape of head and neck oncogenesis, key signaling pathways reported in CXPA were compared with those described in oral squamous cell carcinoma (Table 1).

Table 1 Comparative overview of selected molecular and cellular mechanisms involved in carcinoma ex pleomorphic adenoma (CXPA) and oral squamous cell carcinoma (OSCC). While the initiating oncogenic events differ between the two tumor types, several downstream pathways involved in tumor progression—such as TP53 dysregulation, angiogenesis, and immune microenvironment alterations—show significant biological overlap.

### 3.3. Epigenetic Alterations

Epigenetic dysregulation has emerged as an important contributor to malignant transformation in salivary gland tumors.

Hypermethylation of tumor suppressor genes has been increasingly implicated in the malignant progression of these neoplasms. Epigenetic silencing of *CDKN2A (p16)* and *RASSF1A* has been reported in both benign and malignant salivary gland tumors; however, significantly higher frequencies of promoter hypermethylation have consistently been observed in malignant lesions, suggesting a contributory role in tumor progression and disruption of normal cell cycle regulation [16,18,20].

In contrast, the involvement of *MGMT* promoter methylation in malignant transformation remains controversial. Several studies have failed to demonstrate a statistically significant association between *MGMT* hypermethylation and malignant progression of salivary gland tumors, suggesting that its role may be limited or context-dependent rather than a primary driver of carcinogenesis in this setting [22].

Overall, epigenetic alterations appear to cooperate with genetic changes in driving tumor progression and malignant transformation of benign salivary gland neoplasms.

### 3.4. Histopathological and Clinical Indicators of Malignant Transformation

#### 3.4.1. Clinical Changes

Several clinical features have been consistently associated with malignant transformation of benign salivary gland tumors.

A sudden onset of rapid tumor growth following a prolonged period of clinical stability is considered a key clinical warning sign and has been repeatedly reported in cases of malignant progression, particularly in pleomorphic adenoma [7,42]. Additionally, the development of pain or facial nerve dysfunction, especially in tumors of the parotid gland, raises strong suspicion of malignant involvement and local invasion [30,42]. The presence of regional lymphadenopathy further supports the likelihood of malignant transformation and may indicate locoregional metastatic spread [42].

#### 3.4.2. Histological Changes

Histopathological examination reveals distinct and reproducible features associated with malignant transformation of benign salivary gland tumors.

Malignant lesions demonstrate increased cellular pleomorphism and elevated mitotic activity compared with their benign counterparts, reflecting increased proliferative capacity [29,42]. The presence of tumor necrosis and invasive growth patterns, including perineural and vascular invasion, further supports the diagnosis of malignancy and correlates with aggressive biological behavior [29,42].

In addition, loss of the characteristic biphasic architecture typical of pleomorphic adenoma has been observed, particularly in tumors harboring *HMGA2*-related genetic alterations, where a transition toward more monomorphic histological patterns may occur [12,19].

**Figure 2:**
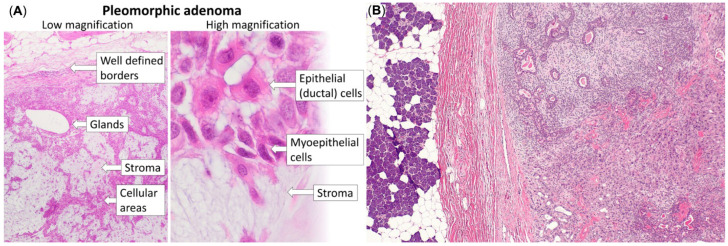
Histopathological comparison between pleomorphic adenoma (**A**) and carcinoma ex pleomorphic adenoma (**B**). (**A**) Pleomorphic adenoma demonstrating characteristic biphasic epithelial and stromal components with well-defined architecture. Image reproduced from Wikimedia Commons (public domain, CC0). (**B**) Carcinoma ex pleomorphic adenoma showing invasive malignant epithelial proliferation beyond the pre-existing adenomatous component. Image reproduced from Wikimedia Commons under the Creative Commons Attribution-ShareAlike 3.0 license (CC BY-SA 3.0). Attribution Section: Panel (**A**) source: Wikimedia Commons. Histopathology of pleomorphic adenoma. Public domain (CC0) (https://commons.wikimedia.org/wiki/Category:Histopathology_of_pleomorphic_adenoma#/media/File:Histopathology_of_pleomorphic_adenoma.png (accessed on 17 February 2026)). Panel (**B**) source: Wikimedia Commons. Carcinoma ex pleomorphic adenoma—low magnification. Licensed under CC BY-SA 3.0 (https://commons.wikimedia.org/wiki/File:Carcinoma_ex_pleomorphic_adenoma_--_low_mag.jpg (accessed on 17 February 2026)). The Figure was created by the combination of the ones above in order to clarify the malignant transformation.

Representative histopathological differences between pleomorphic adenoma and carcinoma ex pleomorphic adenoma are illustrated in Figure 2.

Despite advances in molecular diagnostics, histopathological evaluation remains the cornerstone for the definitive diagnosis of malignant transformation in salivary gland tumors [42].

### 3.5. Angiogenesis and Tumor Microenvironment

Angiogenesis has emerged as a critical mechanism in the malignant progression of salivary gland tumors. Increased expression of CD105 (endoglin), a marker of active neovascularization, together with elevated microvessel density (MVD), has been consistently documented in carcinoma ex pleomorphic adenoma (CXPA) compared with benign pleomorphic adenoma [36,37]. Moreover, a progressive increase in CD105-positive microvessels has been observed along the adenoma–carcinoma sequence, supporting the concept of an angiogenic switch as a key event during malignant transformation [36].

**Figure 3:**
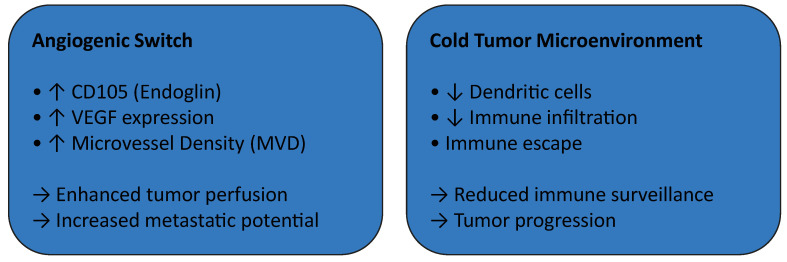
Conceptual model of angiogenic and immune microenvironmental alterations during malignant transformation of benign salivary gland tumors. In carcinoma ex pleomorphic adenoma (CXPA), malignant progression is associated with activation of an angiogenic switch characterized by increased CD105 (endoglin) expression, elevated VEGF signaling, and higher microvessel density (MVD), supporting enhanced tumor perfusion and metastatic potential. Concurrently, tumors exhibit a cold immunological microenvironment, marked by reduced dendritic cell infiltration and limited immune surveillance, facilitating immune escape and tumor progression. ↑ = increase; ↓ = decrease; → = consequence/outcome. This figure was created by the authors for this study.

Angiogenic and immune microenvironmental changes associated with malignant transformation are illustrated in Figure 3.

In parallel, alterations in the tumor immune microenvironment appear to contribute to disease progression. A so-called cold immunological microenvironment, characterized by reduced dendritic cell infiltration and impaired immune surveillance, has been associated with tumor progression and immune escape mechanisms in malignant salivary gland neoplasms [39,40].

**Figure 4:**
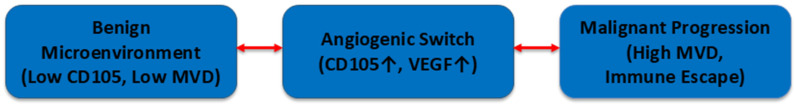
Schematic representation of the angiogenic switch and tumor microenvironment remodeling during malignant progression from benign salivary gland tumors to carcinoma ex pleomorphic adenoma. Benign tumors demonstrate low microvessel density (MVD) and limited CD105 expression. During malignant progression, an angiogenic switch characterized by increased VEGF signaling and CD105-positive neovessels promotes enhanced vascularization. This process is accompanied by immune escape mechanisms and stromal remodeling, supporting carcinoma ex pleomorphic adenoma development. ↑ = increase; the red arrows imply that these mechanisms are interconnected. This figure was created by the authors for this study.

Collectively, these findings highlight the pivotal role of tumor–stroma interactions, integrating angiogenic and immunological pathways, in the pathogenesis of salivary gland carcinogenesis. A schematic representation of the angiogenic switch and tumor microenvironment remodeling during malignant progression is presented in Figure 4.

## 4. Discussion

The present review highlights the complex and multifactorial nature of malignant transformation in benign salivary gland tumors. This process involves the interplay of clinical, histopathological, molecular, and microenvironmental alterations that collectively contribute to tumor progression. Among benign entities, pleomorphic adenoma (PA) consistently emerges as the tumor with the highest risk of malignant transformation, a finding that aligns with long-standing observations in the literature and reinforces its central role in salivary gland carcinogenesis [1,2,3,4,27,28]. The variability in reported transformation rates likely reflects differences in tumor duration, recurrence history, and diagnostic criteria, emphasizing the importance of long-term surveillance and complete surgical excision.

At the molecular level, recurrent genetic rearrangements involving *PLAG1* and *HMGA2* appear to represent early oncogenic events rather than secondary byproducts of malignant transformation. Their persistence in carcinoma ex pleomorphic adenoma (CXPA) supports a multistep tumorigenic model in which initial benign clonal expansion is followed by the progressive accumulation of additional genetic and epigenetic alterations [9,10,11,12]. In particular, *HMGA2*-related alterations have been associated with loss of the classical biphasic morphology and a shift toward more aggressive histological phenotypes, providing a molecular explanation for the morphological continuum observed between PA and CXPA [11].

The acquisition of further genomic abnormalities, including *TP53* mutations and *ERBB2* (HER2/neu) amplification, appears to mark progression toward high-grade malignancy. These alterations are predominantly observed in aggressive CXPA and correlate with invasive growth, increased proliferative activity, and unfavorable clinical outcomes, consistent with observations in other epithelial malignancies [13,14]. This stepwise accumulation of molecular events supports the concept that malignant transformation is not inevitable but occurs once specific biological thresholds are surpassed.

Epigenetic mechanisms further contribute to this process. Hypermethylation of tumor suppressor genes such as *CDKN2A (p16)* and *RASSF1A* has been reported more frequently in malignant salivary gland tumors compared with benign lesions, suggesting that epigenetic silencing contributes to cell cycle dysregulation and tumor progression [15,16,17]. In contrast, evidence regarding *MGMT* promoter methylation remains inconsistent, indicating that its role may be secondary or context-dependent rather than a primary driver of malignant transformation [18].

From a clinical perspective, several features may indicate malignant progression. Sudden acceleration of tumor growth following a prolonged indolent phase, the onset of pain or facial nerve dysfunction, and the presence of regional lymphadenopathy remain important warning signs, particularly in parotid gland tumors [2,19,20,21]. These findings reflect underlying invasive behavior and highlight the importance of early clinical recognition in long-standing or recurrent lesions.

Histopathological examination continues to represent the definitive method for diagnosing malignant transformation. Increased cellular pleomorphism, elevated mitotic activity, tumor necrosis, and invasive growth patterns—including perineural and vascular invasion—are consistently associated with malignant progression and aggressive biological behavior [22,23]. The loss of biphasic morphology in *HMGA2*-altered tumors further illustrates the relationship between molecular alterations and histological phenotype [11].

Beyond tumor cell–intrinsic alterations, the tumor microenvironment plays a decisive role in malignant progression. Increased expression of CD105 (endoglin) and elevated microvessel density (MVD) in CXPA compared with benign PA indicate activation of angiogenic pathways and support the concept of an angiogenic switch during malignant transformation [24,25,26]. Concurrently, the development of a cold immunological microenvironment, characterized by reduced dendritic cell infiltration and impaired immune surveillance, may facilitate immune escape and further tumor progression [29]. These observations highlight the importance of tumor–stroma interactions in salivary gland carcinogenesis.

Despite advances in molecular characterization, several limitations remain. Many available studies are retrospective and involve relatively small cohorts, reflecting the rarity and histological heterogeneity of salivary gland tumors. Furthermore, no standardized molecular diagnostic panel currently exists to reliably predict malignant transformation, limiting the routine clinical application of emerging biomarkers.

Future research should therefore focus on large multicenter prospective studies integrating histopathological evaluation, molecular profiling, and tumor microenvironment analysis. Such approaches may facilitate the identification of robust prognostic biomarkers and support the development of personalized surveillance strategies and targeted therapies aimed at improving outcomes for patients with salivary gland tumors.

## 5. Conclusions

Among benign salivary gland neoplasms, pleomorphic adenoma remains the lesion with the highest documented risk of malignant progression, particularly in long-standing or recurrent cases. In contrast, other benign entities such as Warthin tumors and basal cell adenoma demonstrate a negligible transformation potential.

The present synthesis highlights the pivotal role of recurrent genetic rearrangements, particularly those involving *PLAG1* and *HMGA2*, as early oncogenic events that may persist throughout tumor progression and contribute to architectural disruption and malignant potential. Additional molecular alterations—including *TP53* mutations, *ERBB2* amplification, and epigenetic silencing of tumor suppressor genes—appear to be associated with high-grade malignant transformation and aggressive clinical behavior.

Histopathological evaluation remains the cornerstone for definitive diagnosis, with features such as increased cellular pleomorphism, elevated mitotic activity, necrosis, and invasive growth patterns serving as key indicators of malignant change. Furthermore, emerging evidence underscores the importance of tumor angiogenesis and the immunological microenvironment, with increased CD105 expression, elevated microvessel density, and a cold immune profile contributing to tumor progression and immune escape.

Despite advances in molecular characterization, the absence of a unified diagnostic molecular panel and the heterogeneity of available studies limit the routine clinical application of these biomarkers. Future research should focus on well-designed prospective studies integrating molecular profiling with histopathological and clinical parameters. Such an approach may enable earlier detection of malignant transformation, improved risk stratification, and the development of personalized therapeutic strategies for patients with salivary gland tumors.

## Figures and Tables

**Table 1:** Comparative Molecular Pathways in Carcinoma ex Pleomorphic Adenoma (CXPA) and Oral Squamous Cell Carcinoma (OSCC).

Molecular/Cellular Mechanism	Carcinoma ex Pleomorphic Adenoma (CXPA)	Oral Squamous Cell Carcinoma (OSCC)	Clinical/Biological Significance
Early oncogenic events	*PLAG1* and *HMGA2* rearrangements frequently present in pleomorphic adenoma and retained during malignant transformation	Not typically involved in OSCC	Indicates distinct tumor initiation mechanisms
*TP53* pathway dysregulation	Mutations and loss of p53 activity reported in high-grade CXPA	Very common event in OSCC progression	Promotes genomic instability and tumor progression
EGFR signaling activation	EGFR overexpression and downstream MAPK/PI3K signaling activation reported in malignant salivary gland tumors	EGFR activation frequently observed in OSCC	Drives cell proliferation and tumor growth
HER2 (*ERBB2*) amplification	Amplification particularly reported in aggressive CXPA variants	Rare in OSCC	Associated with aggressive tumor phenotype and potential targeted therapy
Epigenetic alterations	Promoter hypermethylation of tumor suppressor genes (*CDKN2A*, *RASSF1A*) reported during malignant transformation	DNA methylation alterations frequently observed in OSCC	Loss of tumor suppressor activity and deregulated cell cycle
Angiogenesis pathways	Increased VEGF expression and elevated CD105-positive microvessel density reported in CXPA	Strong VEGF-driven angiogenesis in OSCC	Facilitates tumor growth and metastatic potential
Immune microenvironment alterations	Reduced immune surveillance and dendritic-cell-related immune modulation suggested	Tumor immune evasion mechanisms well described in OSCC	Enables tumor progression and immune escape
Tumor invasion mechanisms	Perineural invasion and stromal remodeling reported in CXPA	Common feature in aggressive OSCC	Associated with poor prognosis and local aggressiveness

## Data Availability

No new data were created or analyzed in this study.

## Abbreviations

Abbreviations used in this manuscript:

ADC	Apparent Diffusion Coefficient
APC	Antigen-Presenting Cell
BCA	Basal Cell Adenoma
CXPA	Carcinoma ex Pleomorphic Adenoma
CD105	Endoglin
ERBB2	Erb-B2 Receptor Tyrosine Kinase 2 (HER2/neu)
HMGA2	High-Mobility-Group AT-Hook 2
MVD	Microvessel Density
MGMT	O6-Methylguanine-DNA Methyltransferase
OSCC	Oral Squamous Cell Carcinoma
PA	Pleomorphic Adenoma
PLAG1	Pleomorphic Adenoma Gene 1
RASSF1A	Ras Association Domain Family Member 1
TGF-β	Transforming Growth Factor Beta
TP53	Tumor Protein p53
VEGF	Vascular Endothelial Growth Factor
WHO	World Health Organization
